# Maternal, Valvular and Foetal Outcomes of Pregnancy Following Aortic Valve Replacement

**DOI:** 10.1093/ejcts/ezag137

**Published:** 2026-03-26

**Authors:** Shanti D M Khargi, Pepijn Grashuis, Kevin M Veen, Jolanda Kluin, Jérôme M J Cornette, Mark R Johnson, Jolien W Roos-Hesselink, Johanna J M Takkenberg, Mostafa M Mokhles

**Affiliations:** Department of Cardiothoracic Surgery, Erasmus Medical Centre, Rotterdam,The Netherlands; Department of Cardiothoracic Surgery, Erasmus Medical Centre, Rotterdam,The Netherlands; Department of Cardiothoracic Surgery, Erasmus Medical Centre, Rotterdam,The Netherlands; Department of Cardiothoracic Surgery, Erasmus Medical Centre, Rotterdam,The Netherlands; Department of Obstetrics and Gynaecology, Erasmus Medical Centre, Rotterdam, The Netherlands; Department of Obstetrics and Gynaecology, University of Antwerp, Antwerp, Belgium; Department of Obstetric Medicine, Imperial College London, Kensington, London, United Kingdom; Department of Cardiology, Erasmus Medical Centre, Rotterdam, The Netherlands; Department of Cardiothoracic Surgery, Erasmus Medical Centre, Rotterdam,The Netherlands; Department of Cardiothoracic Surgery, University Medical Centre Utrecht, Utrecht, The Netherlands

**Keywords:** aortic valve replacement, pregnancy, Ross-procedure, bioprosthesis, mechanical valve, homograft

## Abstract

**Objectives:**

To investigate the optimal valve substitute for young women requiring aortic valve replacement (AVR), allowing improved future valve-related outcomes for mother and foetus during pregnancy.

**Methods:**

A systematic search was performed for publications between 1998 and 2025 reporting women experiencing pregnancy after AVR with a pulmonary autograft (Ross-procedure), homograft, bioprosthesis (xenograft), or mechanical valve. Pooled proportions were calculated to determine maternal, valvular and foetal outcomes during pregnancy using generalized linear mixed models.

**Results:**

Thirteen studies reporting 356 pregnancies in 251 women (pooled mean age at pregnancy 29.1 ± 4.8 years) after AVR with a pulmonary autograft (70 women, 119 pregnancies), homograft (73 women, 99 pregnancies), bioprosthesis (37 women, 50 pregnancies), or mechanical valve (71 women, 88 pregnancies) were included. *During pregnancy,* valve-related reintervention in women with a bioprosthesis was 2.7% (95% CI, 0.4-16.9) at 5.1 ± 2.5 years after AVR. This was not observed in women with pulmonary autografts (7.7 ± 4.2 years after AVR) and homografts (4.1 ± 3.3 years after AVR). Reintervention for valve thrombosis (4.9% [95% CI, 1.6-14.0]) and maternal death (1.1% [95% CI, 0.2-7.6]) occurred only in women with mechanical valves (8.1 ± 4.5 years after AVR). Pooled probability of liveborn delivery was 71.7% (95% CI, 59.2-81.6) in women with a mechanical valve, compared to 90.6% (95% CI, 72.4-97.3), 92.3% (95% CI, 56.0-99.1), and 82.9% (95% CI, 53.3-95.4) in women with an autograft, homograft, and bioprosthesis respectively.

**Conclusions:**

Maternal mortality and valve thrombosis during pregnancy occurred only in women with mechanical valves. Although no statistical comparisons were made, pregnancies in women with pulmonary autograft, homograft or bioprosthesis showed acceptable maternal and foetal outcomes. These descriptive findings provide foundations for further investigation of tissue-valve function before, during and after pregnancy, aiming for more support of current guidelines.

## Graphical Abstract

**Figure:**
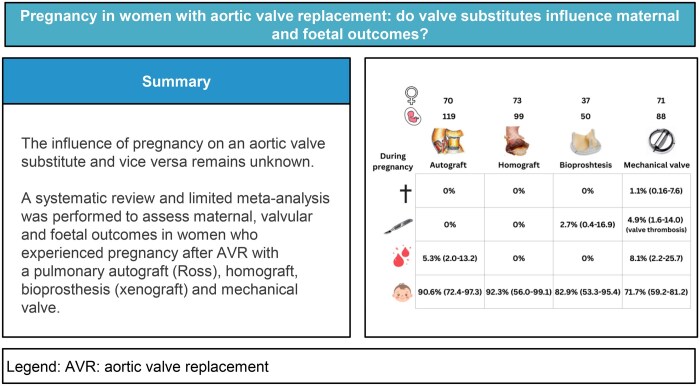

## Introduction

Girls and young women requiring aortic valve replacement (AVR) face an extra challenge when selecting an aortic valve substitute, as future pregnancy must be considered.^1^ A durable valve substitute without the necessity for anticoagulation, allowing both maternal and foetal safety during pregnancy does not exist, making this an unresolved clinical dilemma. Currently, the options encompass a mechanical prosthesis, a pulmonary autograft (Ross-procedure), homograft or biological prosthesis (xenograft).^2^^,^^3^ All categories have distinct advantages and limitations.

A mechanical prothesis is designed to last a lifetime, but lifelong anticoagulation with vitamin-K-antagonists (VKA) is required.^2^ It carries a continuous increased chance of bleeding versus failure to achieve adequate anticoagulation, risking thrombo-embolic complications, including valve thrombosis (VT).^4^ During pregnancy, both of these risks increase further: the maternal hypercoagulable state increases the likelihood of VT, while antepartum and postpartum bleeding risks are also higher due to anticoagulation.^5^ Besides that, teratogenic effects of VKA increase the risk of miscarriage.^6^ Alternative anticoagulation regimens might protect the foetus, at the cost of increased risks of maternal thrombo-embolic events.^7^

Tissue valves do not require anticoagulation. However, they are associated with inevitable valve deterioration, especially in the young, risking earlier reintervention.^8^ During pregnancy, the increase in cardiac output may accelerate valve degeneration, precipitating acute heart failure and possible urgent reintervention.^5^ Nevertheless, recent guidelines recommend biological options in young women who contemplate pregnancy as a class I and IIa recommendation.^1^^,^^2^ Though the level of evidence underlying the high-class recommendations is low (levels B and C), as comprehensive follow-up data on valve-function during and after pregnancy with aortic tissue valves remain limited. Similarly, the optimal anticoagulation strategy for women with mechanical valves during pregnancy and delivery is unknown.

To support guidelines with the currently available evidence, we aim to provide the best possible overview of maternal, valve- and pregnancy-related outcomes in women who experienced pregnancy after AVR, according to valve substitute type. In addition, descriptive outcomes of the different anticoagulation regimens are described for the women with a mechanical valve. With that, we hope to aid the shared decision-making process for women who require AVR in the future.

## Patients and methods

### Protocol and inclusion criteria

This study followed Preferred Reporting Items for Systematic Review and Meta-Analysis (PRISMA, **Table S3**) guidelines, was approved by the Erasmus Medical Ethics Committee (MEC2015-170) and is a sub-study of a meta-analysis registered in PROSPERO with the number CRD42015017041.

Studies were eligible if they included women who conceived after AVR. Studies published after January 1, 1998, which reported ≥10 pregnancies after AVR, and provided at least one clinical outcome of interest (**Table S1**) were included. Case reports and case series with <10 pregnancies were excluded to avoid severe publication bias. Other reasons for exclusion were if (1) patients were selected on pre-existing comorbidities, (2) authors did not specify baseline- and/or outcomes according to valve position, and an AVR-subgroup could not be distilled, (3) the study featured a population in which >20% of the total number of prosthetic valves was not in the aortic position. Multiple valve prostheses in 1 patient or concomitant procedures were not an exclusion criterion. Abstracts, posters, editorials, meta-analyses, and articles without full-text availability or not in English were excluded.

### Search and study selection

On April 12, 2025, a systematic literature search was conducted in Embase, Medline Ovid, Web of Science and Cochrane Library, by a biomedical information specialist in consultation with the study authors (**Supplementary Text S1**). After deduplication, all results were imported into Rayyan.^9^ References published before 1998 were excluded. Remaining references were independently screened for eligibility by 2 reviewers (S.K. and P.G.). In case of overlapping study populations, studies with greater follow-up in patient-years or a larger sample size were preferred for inclusion. Finally, cross-referencing was performed to identify articles missed during the search.

### Data extraction

Two reviewers (S.K. and P.G.) independently extracted data from the included studies into a standardized form in Microsoft-365-Excel. The extracted datasets were cross-checked, and disagreements were resolved by consensus. Extracted variables included baseline patient and operative characteristics, geographical location (high-income country [HIC] and low-middle income country [LMIC]), foetal-, maternal-, cardiac-, and valve-related outcomes (**Table S1**). Events occurring >30-days postpartum onwards are considered long-term. For women with mechanical prostheses, details on anticoagulation regimens during pregnancy were collected.

Valve-related complications such as bleeding (subdivided into obstetric and non-obstetric), thromboembolism, valve thrombosis (VT), structural valve deterioration (SVD), endocarditis, non-valve-related reintervention, heart failure, and valve-related mortality were defined according to the guidelines by Akins et al.^10^ Adverse cardiac/valvular events were considered as pregnancy-related if they occurred during pregnancy or <30-days postpartum.

Definitions for foetal and perinatal complications followed standard terminology defined by Barfield et al.^11^ Miscarriage was defined as pregnancy loss or foetal demise <20 weeks of gestation, and stillbirth for foetal loss ≥20 weeks of gestation. Total pregnancy loss was defined as the total of miscarriages, stillbirths, and terminations of pregnancies. Preterm delivery was defined as livebirth <37 weeks of gestation.

### Statistical analysis

Baseline characteristics were pooled using sample size weighting. Continuous variables are presented for descriptive purposes as means ± SD or medians with interquartile ranges (IQR). Categorical variables are represented as proportions. Subgroup analyses were performed according to valve substitute in the aortic position: Ross-procedure,^3^ homograft, bioprosthesis, and mechanical prosthesis.

For the pooling of proportions, a generalized linear mixed model (GLMM) with a logit link was used, which is the recommended approach for meta-analysis of single proportions, particularly in the presence of zero-event studies.^12^ This method models the binomial data directly and does not require continuity corrections. When possible, pooled outcomes are presented in forest plots. For maternal and valvular outcomes, the number of women was considered the denominator, whereas for foetal outcomes and heart failure during pregnancy, the total number of pregnancies are considered the denominator. All analyses are performed in RStudio using the *meta* and *metadat* packages.^13^ Long-term outcomes with insufficient time-to-event data are summarized descriptively rather than pooled in the meta-analysis. No statistical significance was calculated between subgroups, as they were considered incomparable.

### Quality assessment and sensitivity analysis

The quality of each included study is assessed using the Newcastle-Ottawa Scale for cohort studies.^14^ Sensitivity analyses following the Leave-One-Out principles are performed to determine publication bias.

## Results

### Search results

**Figure 1.**
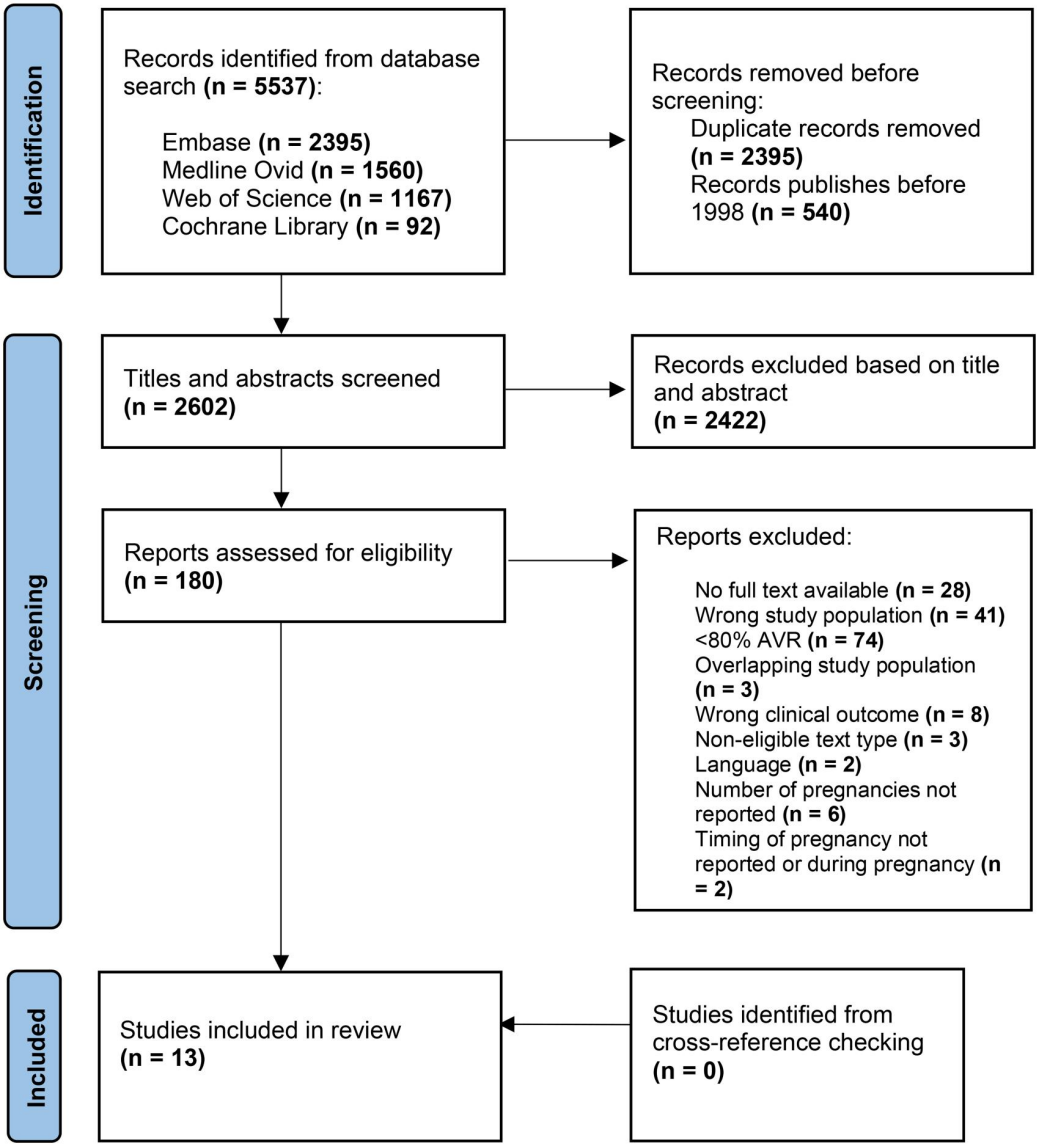
Flowchart of systematic literature search and study selection.

The literature search identified 5537 publications. After deduplication and exclusion of studies published before 1998, abstracts of 2602 publications were screened. Subsequently, 180 articles underwent full-text assessment, after which 13 studies met the inclusion criteria (**Figure 1**). A list of the included studies is provided in **Supplementary Text S2**.

### Baseline characteristics

In total, the included 13 studies reported 251 women and 356 pregnancies. Nine studies provided a pooled mean age at pregnancy of 29.1 ± 4.8 years (**Table 1**). Pooled proportions for the overall study population are presented in **Table 2**. Geographic origin per study is presented in Table S2.

**Table 1.** Pooled Baseline Study- and Patient Characteristics

Study and patient characteristics	Autograft^30–36^	Homograft^32^^,^^37–39^	Bioprosthesis^30^^,^^33^^,^^35^^,^^37^	Mechanical^15^^,^^30^^,^^32^^,^^37^^,^^40^^,^^41^	Total
Number of studies	7	4	4	6	13
Number of patients	70	73	37	71	251
Number of pregnancies	119	99	50	88	356
Pooled mean age at pregnancy, y ± SD	27.5 ± 4.8	30.4 ± 4.6	32.3 ± 5.1	29.9 ± 4.9	29.1 ± 4.9
Pooled mean time from valve replacement to pregnancy, y ± SD	7.7 ± 4.2	4.1 ± 3.3	5.1 ± 2.5	8.1 ± 4.5	6.6 ± 3.7

**Table 2.** Pooled Proportions of Maternal Outcomes—Total Study Population

	Total population	*I* ^2^	*n*
**Maternal outcomes** (%, 95% CI)			
Maternal death	0.2 (0.0-55.5)	0% (*P* = 1.00)	12
Valve-related reintervention (excl. reintervention for VT)	0.5 (0.1-3.4)	0% (*P* = .99)	11
Valve thrombosis (VT)	1.3 (0.18-8.2)	0% (*P* = .98)	11
Other thrombo-embolic event	1.5 (0.48-4.5)	0% (*P* = 1.00)	11
Permanent decrease AI	0.7 (0.0-36.3)	0% (*P* = .74)	7
**Pregnancy outcomes** (%, 95% CI)			
Delivered alive^a^	90.8 (77.4-96.6)	63% (*P* = .00)	13
Miscarriage^a^	3.6 (0.7-16.8)	39% (*P* = .08)	12
Stillbirth^a^	1.3 (0.5-3.4)	0% (*P* = 1.00)	11
Abortion (maternal indication)^a^	1.6 (0.6-4.2)	0% (*P* = 1.00)	9
Bleeding^a^	4.3 (1.7-10.7)	40% (*P* = .09)	10
Heart failure during pregnancy^a^	2.6 (0.9-7.6)	0% (*P* = .99)	10
Pregnancy hypertension^a^	4.4 (2.1-8.9)	0% (*P* = .95)	5
Pre-eclampsia^a^	1.3 (0.3-4.9)	0% (*P* = 1.00)	5
**Foetal outcomes** (%, 95% CI)			
Pre-term birth^a^	8.3 (4.2-15.7)	0% (*P* = .80)	10
Low birth weight^a^	6.2 (2.6-14.0)	20% (*P* = .29)	3
Small for gestational age^a^	5.3 (1.5-16.7)	0% (*P* = .58)	6
Neonatal death^a^	0.8 (0.1-6.5)	0% (*P* = .92)	10

a Calculated per pregnancy.

Abbreviations: *I*^2^, heterogeneity(%), *n*, number of studies reporting the outcome; VT, valve thrombosis.

#### Pulmonary autograft (Ross-procedure)

##### Maternal and valve-related outcomes

Seven studies reported 119 pregnancies in 70 women after a Ross-procedure, deriving from 6 HICs (pooled mean age at pregnancy 27.5 ± 4.8 years; pooled mean interval AVR-pregnancy 7.7 ± 4.2 years).

No maternal deaths, valve-related reinterventions, valve thrombosis, or other thromboembolic events were observed *during pregnancy and within 30-days thereafter* (**Table 3a**). Heart failure occurred in 4.0% (95% CI, 1.3-11.5), of which one woman was described to have heart failure in the third trimester due to recurrent subaortic stenosis. Furthermore, one woman experienced a supraventricular tachycardia during pregnancy.

**Table 3.** Pooled Proportions for Maternal/Valvular (a), Pregnancy(b) and Foetal (c) Outcomes Per Valve Prosthesis

	Ross	*I* ^2^	*n*	Homograft	*I* ^2^	*n*	Bioprosthesis	*I* ^2^	*n*	Mechanical	*I* ^2^	*n*
a. **Maternal outcomes** (%, 95% CI)												
Maternal death	0	0% (*P* = 1.00)	6	0	0% (*P* = 1.00)	3	0	0% (*P* = 1.00)	4	1.1 (0.16-7.6)	0% (*P* = 1.00)	6
Valve-related reintervention (no VT)	0	0% (*P* = 1.00)	5	0	0% (*P* = 1.00)	3	2.7 (0.4-16.9)	0% (*P* = 1.00)	4	0	0% (*P* = 1.00)	6
Valve thrombosis (VT)	0	0% (*P* = 1.00)	6	0	0% (*P* = 1.00)	3	0	0% (*P* = 1.00)	4	4.5 (1.4-13.6)	0% (*P* = .98)	6
Reintervention for VT	–	–	0	–	–	0	–	–	0	4.9 (1.6-14.0)	0% (*P* = .94)	5
Other thrombo-embolic event	0	0% (*P* = 1.00)	6	0	0% (*P* = 1.00)	3	0	0 (*P* = 1.00)	3	3.4 (1.1-10.0)	0% (*P* = .99)	6
Permanent decrease AI	3.9 (0.2-52.8)	0% (*P* = .64)	5	–	–	0	66.7 (15.4-95.7)	NA	1	0	0% (*P* = 1.00)	3
b. **Pregnancy outcomes** (%, 95% CI)												
Delivered alive^a^	90.6 (72.4-97.3)	0% (*P* = .75)	7	92.3 (56.0-99.1)	71% (*P* = .02)	4	82.9 (53.3-95.4)	47% (*P* = .13)	4	71.7 (59.2-81.2)	7% (*P* = .37)	6
Miscarriage^a^	5.8 (2.1-15.0)	0% (*P* = .99)	7	0.15 (0.0-99.0)	0% (*P* = 1.00)	4	9.5 (0.7-61.4)	0% (*P* = .98)	4	21.2 (7.7-46.4)	0% (*P* = .39)	6
Stillbirth^a^	0.2 (0.00-78.7)	0% (*P* = 1.00)	7	0	0% (*P* = 1.00)	3	2.0 (0.3-12.9)	0% (*P* = 1.00)	4	2.7 (0.67-10.0)	0% (*P* = .99)	5
Abortion, maternal indication^a^	0.4 (0.00-61.64)	0% (*P* = 1.00)	6	0	0% (*P* = 1.00)	2	0	0% (*P* = 1.00)	4	2.7 (0.29-20.6)	0% (*P* = 1.00)	5
Bleeding^a^	5.3 (2.0-13.2)	0% (*P* = .99)	5	0	0% (*P* = 1.00)	3	0	0% (*P* = 1.00)	5	8.1 (2.2-25.7)	0% (*P* = .43)	6
Heart failure during pregnancy^a^	4.0 (1.3-11.5)	0% (*P* = 1.00)	5	4.8 (1.6-14.0)	0% (*P* = .98)	3	2.0 (0.28-12.9)	0% (*P* = 1.00)	4	0.5 (0.0-60.0)	0% (P = 1.00)	5
Pregnancy hypertension^a^	1.9 (0.2-19.0)	0% (*P* = 1.00)	3	14.3 (5.5-32.5)	0% (*P* = .99)	2	8.7 (2.2-28.9)	0% (*P* = .74)	2	0	0% (P = 1.00)	5
Pre-eclampsia^a^	0	0% (*P* = 1.00)	4	7.1 (1.8-24.5)	0% (*P* = .99)	2	0	0% (*P* = 1.00)	2	0	0% (*P* = 1.00)	5
c. **Foetal outcomes** (%, 95% CI)												
Pre-term birth^a^	18.4 (11.2-28.7)	0% (*P* = .96)	5	4.7 (1.1-18.2)	0% (*P* = .87)	3	0	0% (*P* = 1.00)	3	11.4 (6.2-19.9)	0% (*P* = .90)	6
Low birth weight^a^	0	NA	1	16.7 (2.3-63.1)	NA	1	4.4 (0.6-25.2)	0% (*P* = .99)	2	4.7 (0.39-38.7)	0% (*P* = 1.00)	3
Small for gestational age^a^	9.1 (2.3-30.3)	0% (*P* = .90)	4	10.7 (3.5-28.4)	0% (*P* = .99)	2	4.4 (0.6-25.2)	0% (*P* = .99)	2	2.3 (0.06-48.2)	0% (*P* = 1.00)	4
Neonatal death^a^	2.1 (0.52-8.0)	0% (*P* = .99)	6	0	0% (*P* = 1.00)	3	0	0% (*P* = 1.00)	4	0.9 (0.01-60.1)	0% (*P* = 1.00)	5

a Calculated per pregnancy.

Abbreviations: *I*^2^, heterogeneity(%); *n*, number of studies reporting the outcome; VT, valve thrombosis.

*Within 6 months post-partum*, 2 other women (2/16, 1.3%/py [95% CI: 0.4-4.0]) developed heart failure. *During further follow-up*, nine women (9/28, 2.7%/py [95% CI: 1.1-6.7]) required reintervention, mostly due to neoaortic root dilatation and autograft regurgitation. Of these, 5/9 underwent concomitant homograft replacement of the right ventricular outflow tract. Other primary reoperation indications were homograft deterioration, recurrent subaortic stenosis as described above, and one acute type A dissection. Progressive aortic dilation after pregnancy was described in another 6 women (6/11), generally without major functional decline within the described follow-up period.

##### Pregnancy and foetal outcomes

**Figure 2.**
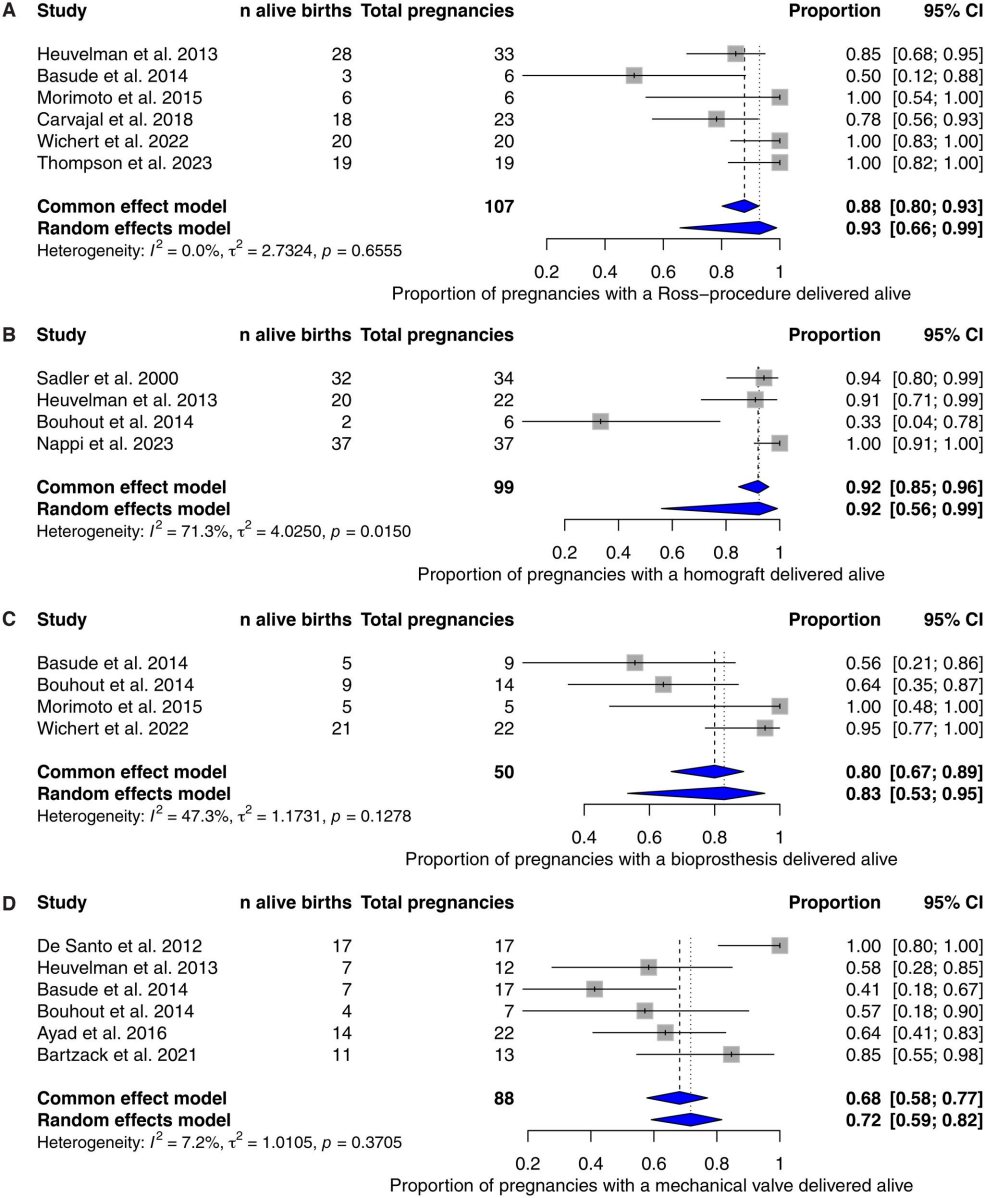
Alive delivery per subgroup (A) Ross, (B) Homograft, (C) Bioprosthesis, and (D) Mechanical.

Pooled livebirth proportion was 90.6% (95% CI: 72.4-97.3, **Table 3b**, **Figure 2A**). Seven pregnancies were terminated (5.9% [95% CI: 2.8-11.8], of which one was for maternal health issues (0.4% [95% CI: 0.0-61.6]). Obstetric haemorrhage occurred in 5.3% (95% CI: 2.0-13.2), including 2 antepartum placental abruptions (26 and 34 gestational age). Pre-term birth occurred in 18.4% (95% CI: 11.2%-28.7%, **Table 3**); 2 cases were due to placental abruption as described above, with one resulting in neonatal death. A second neonatal death occurred in a neonate of a mother on VKA for protein-C deficiency.

#### Homograft

##### Maternal and valve-related outcomes

Four studies reported 99 pregnancies in 73 women after homograft AVR, deriving from 5 HICs (pooled mean maternal age 30.4 ± 4.6 years; pooled mean interval AVR–pregnancy 4.1 ± 3.3 years).

No maternal death, valve failure, reinterventions, valve thrombosis, thromboembolisms or haemorrhage were observed *during pregnancy or within 30-days postpartum* (**Table 3a** and **b**). Heart failure occurred in three women: 4.8% (95% CI: 1.6–14.3), none with evidence of involvement of the valve substitute.

*During follow-up >30 days post-partum*, one study reported 17 deaths in 37 women up to 20 years after AVR, including 4 valve-related deaths, though without further details.

##### Pregnancy and foetal outcomes

The livebirth proportion was 92.3% (95% CI: 56.0-99.1, **Table 3**, **Figure 2B**). No cases of therapeutic abortion, stillbirth, or neonatal death were reported. Miscarriage occurred in 4 pregnancies, all in one patient due to alloimmunization.

#### Bioprosthesis (xenografts)

##### Maternal valve-related outcomes

Four studies reported 50 pregnancies in 37 women after bioprosthetic AVR, deriving from 5 HICs (pooled maternal mean age 32.3 ± 5.1 years; pooled mean interval AVR-pregnancy 5.1 ± 2.5 years).

No maternal deaths, valve thrombosis, thromboembolisms or postpartum haemorrhage occurred *during pregnancy or within 30-days thereafter*. Two women deteriorated from NYHA class II to III due to progressive SVD; one required repeat valve replacement during pregnancy at 23 weeks but experienced a stillbirth afterwards.

One mother died around *first 6 weeks post-partum*, at nine years after valve implantation, of an unknown cause. Two women (2/22, 0.7%/py [95% CI: 0.1-5.0]) developed heart failure within 6 months post-partum, without requiring reintervention. In addition, 3 women required reoperation (3/46, 6.61%/py [95% CI: 2.1-20.5]) due to bioprosthesis-failure during a follow-up, of which 2 within 1 year after a second pregnancy.

##### Pregnancy and foetal outcomes

Pooled livebirth proportion was 82.9% (95% CI: 53.3-95.4, **Table 3b**, **Figure 2C**), and stillbirth was 2.0% (95% CI: 0.3-12.9). No abortions or neonatal deaths were reported.

#### Mechanical prosthesis

##### Maternal and valve-related outcomes

Six studies reported 88 pregnancies in 71 women after mechanical AVR, deriving from 6 HICs and 1 LMIC (pooled mean age 29.9 ± 4.9 years; pooled mean interval AVR–pregnancy 8.1 ± 4.5 years).

**Figure 3.**
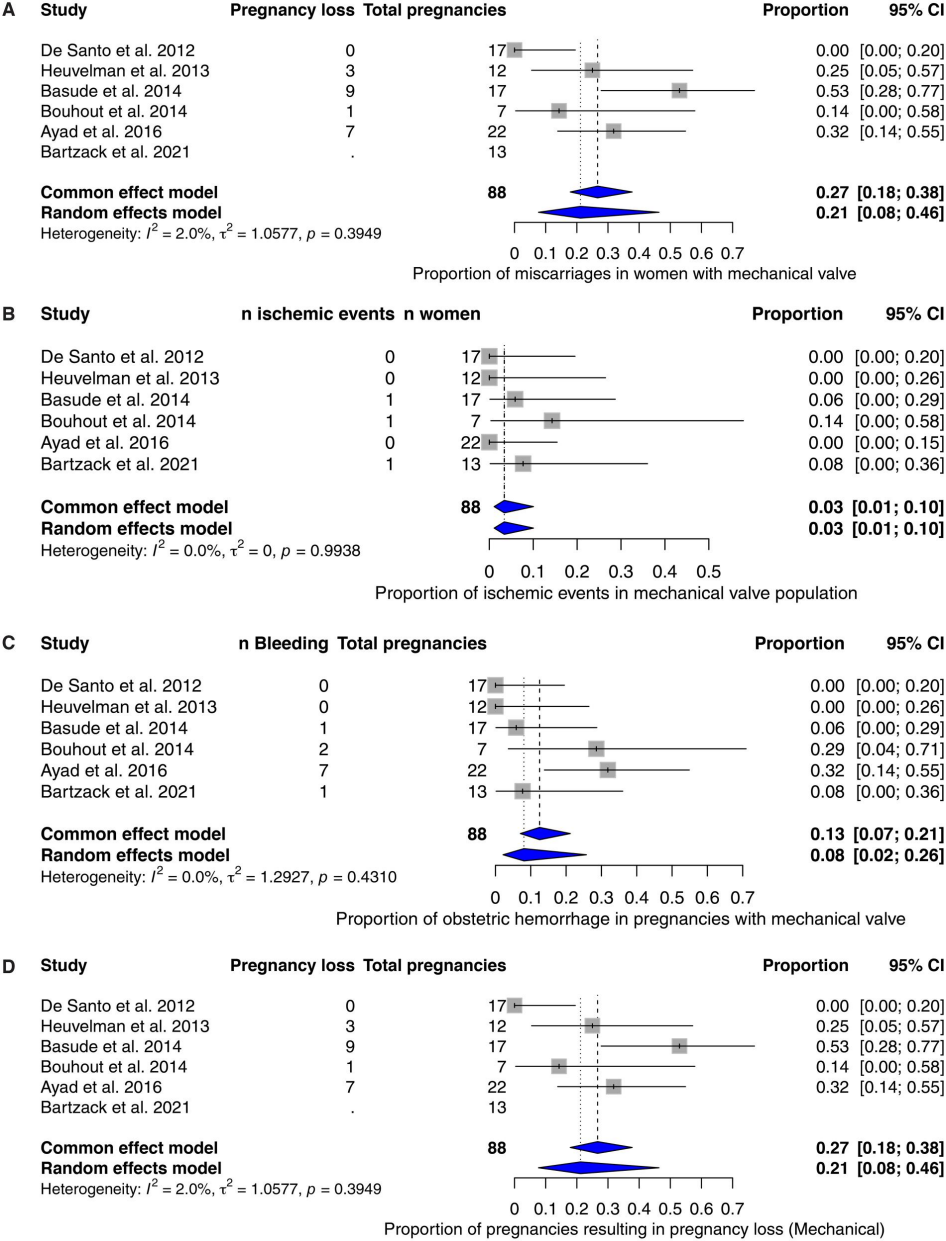
Forest plots mechanical subgroup (A) Valve thrombosis, (B) Other ischemic events, (C) Obstetric hemorrhage, and (D) Miscarriage.

Maternal mortality *during pregnancy* was 1.1% (95% CI: 0.16-7.6). This was due to valve thrombosis in a 22-year-old woman with a tilting-disc valve, which she received 17 years before conception. Valve thrombosis occurred in 2 additional cases (total proportion 4.46%; 95% CI: 1.4-13.6, **Figure 3A**), both patients were successfully reoperated. Three other thrombo-embolic events occurred during pregnancy (3.4%, 95% CI: 1.1-10.0, **Figure 3B**), all resulting in pregnancy losses.

Obstetric haemorrhage occurred in 11 pregnancies, with a pooled proportion of 8.1% (95% CI: 2.2-25.8, **Figure 3C**). Heart failure was reported in three patients during pregnancy (0.5% [95% CI: 0.0-60.0]). These cases were attributed to patient-prosthesis mismatch, valve thrombosis and one unknown cause.

One maternal death occurred *2 years postpartum* due to declining function of the left ventricle and anticoagulation non-compliance. Another woman developed myocardial infarction 3 months postpartum, despite resuming warfarin. She was at a sub-therapeutic International Normalized Ratio (INR) of 1.5.

##### Pregnancy and foetal outcomes

Miscarriage occurred in 20/88 pregnancies (21.2%, 95% CI: 7.7-46.4, **Figure 3D**) and termination of pregnancy due to maternal indications occurred in four cases (2.7%, 95% CI: 0.29-20.6). No cases of alive-born coumarin embryopathy were reported in the liveborn neonates.

##### Anticoagulation regimen

Five studies provided detailed anticoagulation strategies in 66 women, reporting the outcomes of seven different regimens (22 pregnancies are insufficiently reported). Maternal post-partum bleeding in relationship to anticoagulation regimen was scarcely reported. A summary is presented in **Table 4**.

**Table 4.** Descriptive Outcomes of Pregnancies in Women with Mechanical Valve; According to Anticoagulation Regimen

	*n* women	Maternal outcomes	Fetal outcomes
Warfarin throughout pregnancy	32	Valve thrombosis (VT): 1/32 at gestational week 33 with sub-therapeutic INR. Reoperation 5 weeks post-partum	Total pregnancy loss: 6/32
Warfarin <5 mg	19		19/19 livebirths
Warfarin 4-10 mg	13		7/13 livebirths 6/13 miscarriages
LMWH during 1st trimester → warfarin until week 36 → LMWH before delivery	14	Thrombo-embolic adverse event: 3/14 VT at gestational week 11 → reintervention during pregnancy Myocardial infarction first trimester Transient ischaemic attack first trimester	Delivered alive: 8/14 (of which one in mother A) Total pregnancy loss: 6/14 1 still birth 1 termination (in mother B) 4 miscarriages after switch to VKA (of which one confirmed anticoagulation embryopathy in mother C)
UFH during the first trimester → warfarin until week 36 → UFH before delivery	8	Uncomplicated	Uncomplicated
LMWH throughout pregnancy	6	Maternal death due to VT and emergency surgery: 1/6	Delivered alive: 3/6 Pregnancy loss: 3/6 Miscarriage: Termination: 1 Stillbirth: 1 (in mother with VT who died)
UFH throughout pregnancy	3	Stroke: 1/3 Late post-partum bleeding: 1/3	Delivered alive: 1/3 Terminations: 2/3 (1 after maternal stroke)
Low-dose aspirin + LMWH	2	Uncomplicated	Uncomplicated
LMWH + VKA	1	Uncomplicated	Uncomplicated

Abbreviations: LMWH, low-molecular-weight-heparin; UFH, unfractionated heparin; VKA, vitamin-K-antagonist; VT, valve thrombosis.

#### Sensitivity analysis and quality assessment

Sensitivity analyses following the leave-one-out principle revealed no substantial changes in the outcomes. Results of the exclusion of the only non-HIC are presented in **Supplementary Table S6** and **Figure S1A-S1D**.^15^ The Newcastle-Ottawa Scale quality assessment of the included cohort studies is presented in **Supplementary Table S7**.

## Discussion

This systematic review summarizes maternal, valve-related and pregnancy outcomes in women with different types of aortic valve substitutes, as well as outcomes of various anticoagulation strategies in those with mechanical valves. Despite limited and descriptive data only, our findings present acceptable maternal and foetal outcomes of pregnancy in women with aortic tissue valves. Our results follow the trend that women with a mechanical valve have lower proportions of livebirth, which is likely associated with anticoagulation.^6^^,^^16^^,^^17^ Maternal mortality and thrombo-embolic events were observed in the women with a mechanical valve only. The only subgroup encompassing an LMIC was in the mechanical valve subgroup, while all women with tissue valves originated from HICs.

**Figure 4.**
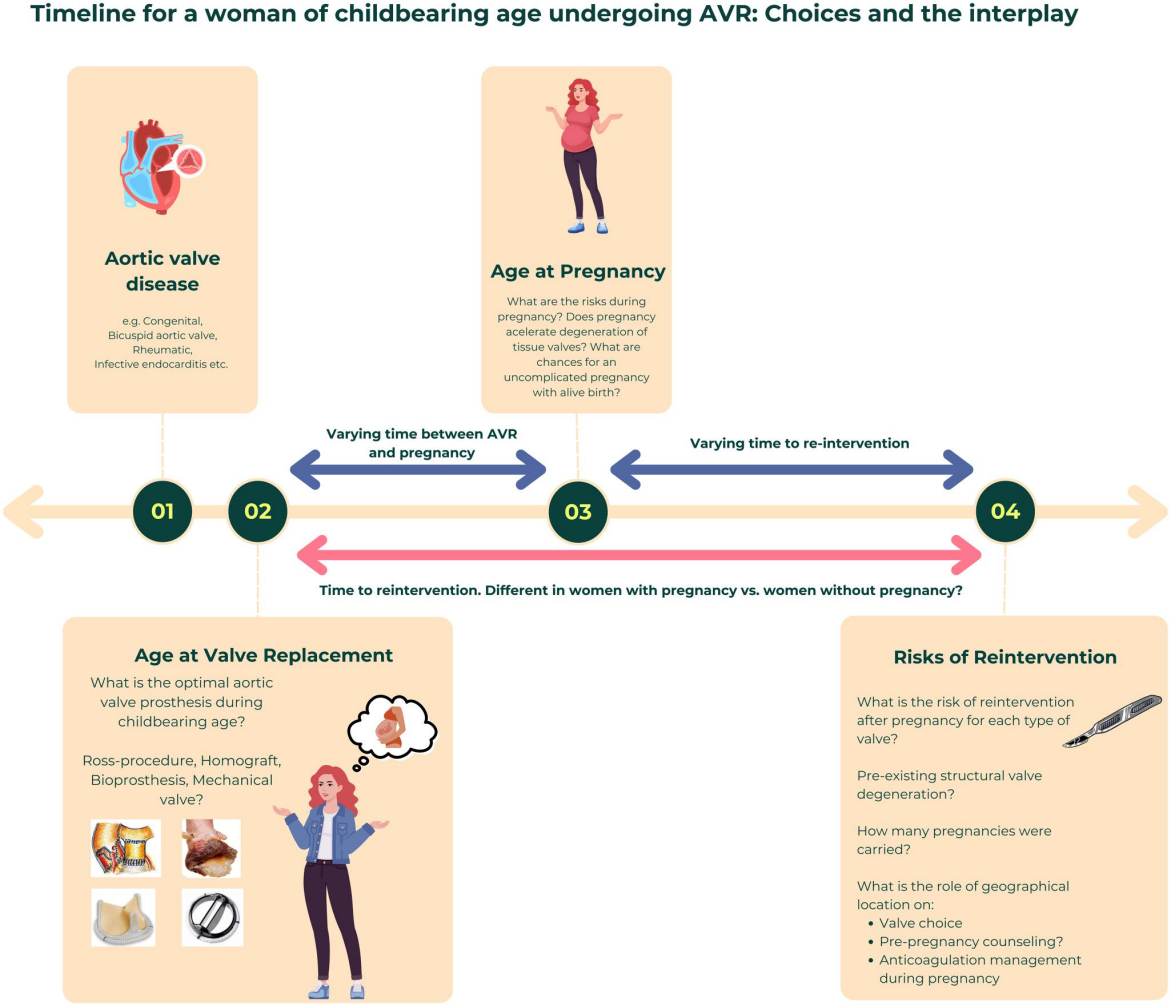
Lifetime timeline for women requiring valve replacement.

Current European guidelines advice a tissue prosthesis for women of childbearing age who wish to conceive in the future—despite the risk of early reintervention—and are not worldwide applicable. Furthermore, lifetime data of these women, including haemodynamic performance of the valve-prosthesis pre- and post-conception, is lacking and can vary per individual (**Figure 4**). Therefore, this meta-analysis is not only a descriptive summary of valvular outcomes, but it is also a worldwide call for more and detailed registration on the long-term behaviour of tissue valves in all young women: from the moment that the valve is implanted until the moment that the valve is explanted, regardless of whether a pregnancy was carried. With that, in the future, we aim to assess the theory of accelerated valve deterioration due to haemodynamic strain during pregnancy. This information is necessary to council future girls and women who require AVR and to upgrade the level of evidence to the current class I recommendations in the guidelines.^1^

### SVD in tissue valve prostheses

SVD remains the major limitation of tissue valves. It develops progressively over time, more rapidly in younger patients and could be present at conception already.^8^ Pregnancy-related haemodynamic strain may accelerate this process, potentially leading to acute heart failure during pregnancy or earlier reintervention.^5^ In women with a tissue valve, there were no maternal deaths or thrombotic events. Valve reintervention during pregnancy was uncommon and occurred only in bioprostheses. One woman required reintervention shortly after pregnancy, due to recurrent subvalvular stenosis and subsequent heart failure during the third trimester. No re-interventions occurred in homografts, though AVR-to-pregnancy intervals were relatively short. It confirms earlier Danish findings that women with aortic tissue valves have appropriate pregnancy toleration.^16^ Still, heart failure remains a concern, reflected in this study. Due to limited follow-up after pregnancy and heterogeneous reporting, valve function and reintervention rates in the years after pregnancy were difficult to pool reliably (**Table S5**). To give the best possible overview of the post-partum valve durability, the available results were descriptively stated.

Nevertheless, tissue valve-related outcomes are determined by an interplay of maternal- and valve-related factors such as age at AVR, age at conception, pre-conception SVD and parity (**Figure 4**). To gain quantitative insights, standardized individual patient-documentation with echocardiographic repeated measurements of each woman are necessary, starting the moment that AVR is performed. Furthermore, socio-economic-status and geographical differences also contribute: tissue valves are more frequently implanted in younger women in HICs, while in LMIC-settings, mechanical valves remain predominant due to durability concerns and compromised healthcare accessibility and costs.^18^

**Figure 4** illustrates the importance of comprehensive holistic pre-operative counselling of young girls and their parents, involving extensive explanation of the impact of a future pregnancy on the underlying heart disease, valve substitute and possible anticoagulation, when the valve substitute options are presented. Later in life, pre- and peri-conceptional valve assessment and counselling remain important to assess valvular performance, aiming for increased favourable outcomes for mother and foetus in all parts of the world.

### Mechanical valves and anticoagulation

Balancing adequate anticoagulation versus bleeding remains a continuous challenge for women with a mechanical valve, especially during the hypercoagulable state of pregnancy. Valve thrombosis and other thromboembolic events during pregnancy were only observed in women with a mechanical valve. They occurred in women on different anticoagulation regimens: VKA (sometimes sub-therapeutic INR), UFH followed by VKA, and LMWH throughout pregnancy. INR and anti-Xa levels were not consistently reported. Above all, close monitoring of these levels has not been proven to substantially improve outcomes or prevent thrombo-embolic adverse events.^18^ On the other side of the spectrum, concerns remain about pregnancy on anticoagulation, showing substantially higher bleeding risks, especially during delivery.^19^

Furthermore, in current literature, anticoagulation-switch-moments are described as high-risk moments for the development of VT.^20^ We did not see a specific trend of occurrence of thrombo-embolic event in relationship to a recent switches. Though three ischaemic events occurred during LMWH use in the first trimester, leading to pregnancy loss after switching to VKA. It also underscores the persistent maternal thrombo-embolic risks of heparin (LMWH and UFH) during pregnancy, and addresses concerns raised in earlier studies.^16^^,^^17^^,^^20^^,^^21^ These issues emphasize the need for alternative anticoagulation regimens for women undergoing pregnancy. Suggestions have been made with aspirin in combination with low-dose VKA for women with low-bleeding risks, which combines the theory that low-dose VKA allows safe pregnancy on one side and substantially decreases strokes, VT and mortality on the other side.^7^^,^^19^^,^^22^ We report one woman on this regimen, who experienced an uncomplicated pregnancy.

Recently, a mechanical valve in the low-flow mitral position was described as a risk factor for VT during pregnancy.^18^ Contrastingly, when placing these outcomes next to the outcomes of our previous meta-analysis on pregnancy in women with *mitral* valve prostheses, we found comparable risks for VT in mechanical valves: 4.8% in the mitral versus 4.5% in the aortic population.^23^ When VT occurs during pregnancy, it often requires immediate reoperation. For this specific group, mechanical mitral valve-prosthesis reoperation was identified as a serious risk factor for mortality and adverse events for the mother and the foetus in utero.^24^

With regard to foetal and obstetric outcomes, our data showed acceptable foetal livebirth proportions for women with tissue valves, while the absolute risk of pregnancy loss in mechanical valve showed a trend of poorer outcomes, although not statistically confirmed. These findings align with prior reports, such as the Registry of Pregnancy and Cardiac Disease (ROPAC)-registry, which showed higher foetal loss with mechanical valves (27.2%) compared to tissue valves (5.5%), regardless of the type of anticoagulation used in the first trimester of pregnancy.^18^^,^^25^ Surgical reintervention during pregnancy, especially during the first trimester, was identified as an important risk factor for foetal loss.^24^ Furthermore, our data suggest dose-dependent VKA-effects, an observation that is also mentioned in current guidelines: women receiving <5 mg warfarin daily all had live births, while women on higher doses of warfarin showed considerable pregnancy losses.^1^

### Geographic location

Most studies derive from HICs (Europe and North America). These results could overestimate real-world data of pregnancy in women with aortic substitutes, as data from LMICs are lacking or inconsistently reported. The sensitivity analysis, in which we excluded the LMIC, showed no substantial changes in maternal and foetal outcomes.

Investigators from the Indian Madras Medical College Pregnancy and Cardiac Registry (M-PAC) report that Indian women with mechanical valves had substantially higher pregnancy loss, maternal bleeding and thrombo-embolic events compared to ROPAC-patients.^26^ On one side, this trend could be explained by the geographical location that could have an impact on the choice of valve: LMIC tend to favour a mechanical valve, due to underlying heart disease, lifelong durability while accessibility to repeat valve-replacement might be lower and costly.^27^ On the other side, pregnancy counselling and management of anticoagulation during pregnancy might not be well organized, or knowledge on the management in LMICs might be compromised.^28^ Therefore, the M-PAC authors call for a universal cardio-obstetric team approach, aiming to exchange knowledge and strengthen pre-operative and pre-conception counselling in LMICs.^26^

To improve future research in high-, middle-, and low-income countries, standardized reporting of maternal, foetal, and valve outcomes is essential. Large prospective registries such as ROPAC-III and M-PAC registry have already advanced this field. Ideally, longitudinal cohorts follow women from valve replacement throughout life. Such data could allow predictive modelling of pregnancy feasibility, optimal timing after tissue-valve implantation, and expected valve durability, thereby supporting clinical and shared decision-making. Eventually, these data will ensure generalizable evidence in guidelines for young girls and women worldwide.

### Strengths and limitations

This review represents the largest synthesis of maternal, valvular, and foetal outcomes of pregnancy after AVR per subtype. These data are of increasing value as more women with congenital or acquired heart-valve disease reach childbearing age.

Several limitations must be acknowledged for adequate appraisal of the data. Firstly, interpretation and clinical applicability of the results is very limited due to small, heterogeneous, and highly selected populations. To give an overview of the data as best as possible, we did perform a meta-analysis for peri-pregnancy outcomes and post-partum outcomes if feasible. Though, pooling of rare events in small cohorts was statistically difficult, which sometimes led to wide confidence intervals and unreliable *I*^2^-values, reflecting a lack of statistical power instead of homogeneity.

Secondly, selection bias and immortal time bias play a role in this manuscript. For example, women deemed fit for pregnancy might be more likely to become pregnant, as unfit women are advised against pregnancy. Moreover, some included studies excluded early pregnancy loss, risking an underreporting of miscarriages and/or stillbirths. Publication bias is also present in our study, especially in relationship to geographical location. Other forms of publication bias could not be assessed using funnel plots, as the absolute risks presented in this overview, are associated with methodological limitations for a meaningful interpretation of the funnel plot.^29^

Maternal and valve-specific details remain underreported, such as time between index-operation and pregnancy as well as timing of re-interventions during follow-up after pregnancy. This restricted haemodynamic assessments, and evaluation of valve function over time. Consequently, we could not answer the question whether pregnancy influences maternal and foetal outcomes and the theory of accelerated valve deterioration during pregnancy. All in all, these limitations emphasize the need for further data-collection, thorough monitoring and detailed uniform reporting of maternal and valvular function from the moment that AVR is performed.

Despite these limitations, this review integrates valve-specific outcomes in pregnant women across 4 prosthesis types, with acceptable maternal, valvular, and foetal outcomes during pregnancy, although no statistical comparisons could be made. A persistent need remains to achieve more evidence-based pre-AVR and pre-pregnancy counselling for women of childbearing age who require AVR.

## Conclusion

The dilemma for an optimal aortic valve for women with a pregnancy wish remains a difficult balance, when aiming for maternal *and* foetal safety. Maternal mortality, valve thrombosis, and other thrombo-embolic events during pregnancy occurred only in women with mechanical valves. Pregnancies after AVR in women with pulmonary autograft, homograft, or bioprosthesis showed acceptable maternal and foetal outcomes. Long-term effects of pregnancy on overall durability in tissue valves and vice versa remain uncertain. More evidence, with detailed follow-up on valvular function, is necessary to answer the question of whether pregnancy accelerates biological valve degeneration. With that, we hope to determine optimal timing for a safe pregnancy with a tissue valve for the mother and foetus in the future. This will help to council future women requiring AVR with a possible wish to conceive. For now, shared decision-making remains essential in young women requiring AVR, with careful weighing of individual risks and preferences.

## Supplementary Material

ezag137_Supplementary_Data

## Data Availability

The extracted data used for the analysis in this manuscript are available upon request to the corresponding author.

## Abbreviations

AVR: aortic valve replacement
CI: confidence interval
HIC: high-income country
INR: International Normalized Ratio
LMIC: low-middle-income country
LMWH: low-molecular-weight heparin
SVD: structural valve deterioration
UFH: unfractionated heparin
VKA: vitamin-K-antagonist
