# Supplementary material for: Maternal, Valvular and Foetal Outcomes of Pregnancy Following Aortic Valve Replacement
Source: Eur J Cardiothorac Surg. 2026 Mar 26;68(4):ezag137. doi: 10.1093/ejcts/ezag137 (PMC13070675; doi:10.1093/ejcts/ezag137)

# Supplementary materials

## Text 1. Literature search terms

### embase.com

('heart valve replacement'/exp OR 'heart valve prosthesis'/exp OR ((implantation/de OR bioprosthesis/de) AND 'heart valve'/exp) OR 'prosthetic valve thrombosis'/de OR (((valv* OR tricuspid OR mitral OR aort* OR pericard*) NEAR/6 (replace* OR substitut* OR prosthe* OR bioprosth* OR prothe* OR bioproth* OR artificial* OR implant* OR mechanic* OR metal OR biological*)) OR (ross NEXT/1 (procedure* OR graft* OR autograft* OR homograft* OR xenograft*))):ab,ti) AND ('pregnancy'/exp OR 'pregnant woman'/exp OR 'pregnancy outcome'/exp OR 'fetus death'/exp OR obstetrics/exp OR 'pregnancy disorder'/exp OR 'perinatal mortality'/exp OR 'maternal mortality'/exp OR 'obstetric procedure'/de OR 'obstetric delivery'/exp OR 'birth weight'/exp OR 'prematurity'/exp OR 'small for date infant'/exp OR 'neonatal intensive care unit'/exp OR (pregnan* OR obstetric* OR stillbirth* OR still-birth* OR stillborn* OR still-born* OR abortion* OR ((perinatal* OR prenatal* OR postnatal* OR peri-natal* OR pre-natal* OR post-natal* OR maternal* OR postpart* OR intrapart* OR antepart* OR post-part* OR intra-part* OR ante-part* OR fetus OR fetal OR foetus OR foetal OR neonat* OR newborn* OR gestation* OR baby OR babies OR maternal*) NEAR/6 (mortalit* OR death OR hemmorrhag* OR bleeding* OR outcome*)) OR obstetric* OR cesarean* OR caesarean* OR 'birth weight' OR birthweight OR Preterm OR Pre-term OR (premature* NEAR/3 (birth OR childbirth* OR labor OR labour OR deliver*)) OR (small NEAR/3 (date OR gestation* OR age)) OR sga OR lbw OR vlbw OR elbw OR (neonatal NEAR/3 intensive NEAR/3 care) OR nicu OR nicus):ab,ti) NOT ([animals]/lim NOT [humans]/lim) NOT ([Conference Abstract]/lim) AND [english]/lim

### Medline Ovid

(Heart Valve Prosthesis Implantation / OR Heart Valve Prosthesis / OR ((Bioprosthesis /) AND exp Heart Valves/) OR (((valv* OR tricuspid OR mitral OR aort* OR pericard*) ADJ6 (replace* OR substitut* OR prosthe* OR bioprosth* OR prothe* OR bioproth* OR artificial* OR implant* OR mechanic* OR metal OR biological*)) OR (ross ADJ (procedure* OR graft* OR autograft* OR homograft* OR xenograft*))).ab,ti.) AND (exp Pregnancy/ OR Pregnant Women/ OR exp Pregnancy Outcome/ OR Perinatal Death/ OR Fetal Death/ OR Obstetrics/ OR Perinatal Mortality/ OR Maternal Mortality/ OR exp Delivery, Obstetric / OR exp Birth Weight/ OR Infant, Premature/ OR Infant, Small for Gestational Age/ OR Intensive Care Units, Neonatal/ OR (pregnan* OR obstetric* OR stillbirth* OR still-birth* OR stillborn* OR still-born* OR abortion* OR ((perinatal* OR prenatal* OR postnatal* OR peri-natal* OR pre-natal* OR post-natal* OR maternal* OR postpart* OR intrapart* OR antepart* OR post-part* OR intra-part* OR ante-part* OR fetus OR fetal OR foetus OR foetal OR neonat* OR newborn* OR gestation* OR baby OR babies OR maternal*) ADJ6 (mortalit* OR death OR hemmorrhag* OR bleeding* OR outcome*)) OR obstetric* OR cesarean* OR caesarean* OR birth weight OR birthweight OR Preterm OR Pre-term OR (premature* ADJ3 (birth OR childbirth* OR labor OR labour OR deliver*)) OR (small ADJ3 (date OR gestation* OR age)) OR sga OR lbw OR vlbw OR elbw OR (neonatal ADJ3 intensive ADJ3 care) OR nicu OR nicus).ab,ti.) NOT (exp animals/ NOT humans/) AND english.la.

### Web of Science

TS=(((((valv* OR tricuspid OR mitral OR aort* OR pericard*) NEAR/5 (replace* OR substitut* OR prosthe* OR bioprosth* OR prothe* OR bioproth* OR artificial* OR implant* OR mechanic* OR metal OR biological*)) OR (ross NEAR/1 (procedure* OR graft* OR autograft* OR homograft* OR xenograft*)))) AND ((pregnan* OR obstetric* OR stillbirth* OR still-birth* OR stillborn* OR still-born* OR abortion* OR ((perinatal* OR prenatal* OR postnatal* OR peri-natal* OR pre-natal* OR post-natal* OR maternal* OR postpart* OR intrapart* OR antepart* OR post-part* OR intra-part* OR ante-part* OR fetus OR fetal OR foetus OR foetal OR neonat* OR newborn* OR gestation* OR baby OR babies OR maternal*) NEAR/5 (mortalit* OR death OR hemmorrhag* OR bleeding* OR outcome*)) OR obstetric* OR cesarean* OR caesarean* OR "birth weight" OR birthweight OR Preterm OR Pre-term OR (premature* NEAR/2 (birth OR childbirth* OR labor OR labour OR deliver*)) OR (small NEAR/2 (date OR gestation* OR age)) OR sgaOR lbw OR vlbw OR elbw OR (neonatal NEAR/2 intensive NEAR/2 care) OR nicu OR nicus)))

### Cochrane Library

((((valv* OR tricuspid OR mitral OR aort* OR pericard*) NEAR/6 (replace* OR substitut* OR prosthe* OR bioprosth* OR prothe* OR bioproth* OR artificial* OR implant* OR mechanic* OR metal OR biological*)) OR (ross NEXT/1 (procedure* OR graft* OR autograft* OR homograft* OR xenograft*))):ab,ti) AND ((pregnan* OR obstetric* OR stillbirth* OR still-birth* OR stillborn* OR still-born* OR abortion* OR ((perinatal* OR prenatal* OR postnatal* OR peri-natal* OR pre-natal* OR post-natal* OR maternal* OR postpart* OR intrapart* OR antepart* OR post-part* OR intra-part* OR ante-part* OR fetus OR fetal OR foetus OR foetal OR neonat* OR newborn* OR gestation* OR baby OR babies OR maternal*) NEAR/6 (mortalit* OR death OR hemmorrhag* OR bleeding* OR outcome*)) OR obstetric* OR cesarean* OR caesarean* OR 'birth weight' OR birthweight OR Preterm OR Pre-term OR (premature* NEAR/3 (birth OR childbirth* OR labor OR labour OR deliver*)) OR (small NEAR/3 (date OR gestation* OR age)) OR sga OR lbw OR vlbw OR elbw OR (neonatal NEAR/3 intensive NEAR/3 care) OR nicu OR nicus):ab,ti)

## Supplementary Text S2: References included for final analysis

1. Thompson SE, Prabhakar CRK, Creasey T, Stoll VM, Gurney L, Green J, et al. Pregnancy outcomes in women following the Ross procedure. Int J Cardiol. 2023;371:135-9.
2. Wichert-Schmitt B, Grewal J, Malinowski AK, Pfaller B, Losenno KL, Kiess MC, et al. Outcomes of Pregnancy in Women With Bioprosthetic Heart Valves With or Without Valve Dysfunction. J Am Coll Cardiol. 2022;80(21):2014-24.
3. Basude S, Trinder J, Caputo M, Curtis SL. Pregnancy outcome and follow-up cardiac outcome in women with aortic valve replacement. Obstet Med. 2014;7(1):29-33.
4. Carvajal HG, Lindley KJ, Shah T, Brar AK, Barger PM, Billadello JJ, Eghtesady P. Impact of pregnancy on autograft dilatation and aortic valve function following the Ross procedure. Congenit Heart Dis. 2018;13(2):217-21.
5. Heuvelman HJ, Arabkhani B, Cornette JM, Pieper PG, Bogers AJ, Takkenberg JJ, Roos-Hesselink JW. Pregnancy outcomes in women with aortic valve substitutes. Am J Cardiol. 2013;111(3):382-7.
6. Morimoto K, Hoashi T, Kagisaki K, Yoshimatsu J, Shiraishi I, Ichikawa H, et al. Impact of Ross Operation on Outcome in Young Female Adult Patients Wanting to Have Children. Circ J. 2015;79(9):1976-83.
7. Yap SC, Drenthen W, Pieper PG, Moons P, Mulder BJ, Klieverik LM, et al. Outcome of pregnancy in women after pulmonary autograft valve replacement for congenital aortic valve disease. J Heart Valve Dis. 2007;16(4):398-403.
8. Bouhout I, Poirier N, Mazine A, Dore A, Mercier LA, Leduc L, El-Hamamsy I. Cardiac, obstetric, and fetal outcomes during pregnancy after biological or mechanical aortic valve replacement. Can J Cardiol. 2014;30(7):801-7.
9. Nappi F, Nenna A, Spadaccio C, Avtaar Singh SS, Almazil A, Acar C. The Use of the Cryopreserved Aortic Homograft for Aortic Valve Replacement: Is It Still an Option? J Cardiovasc Dev Dis. 2023;10(6).
10. Sadler L, McCowan L, White H, Stewart A, Bracken M, North R. Pregnancy outcomes and cardiac complications in women with mechanical, bioprosthetic and homograft valves. BJOG. 2000;107(2):245-53.
11. Ayad SW, Hassanein MM, Mohamed EA, Gohar AM. Maternal and Fetal Outcomes in Pregnant Women with a Prosthetic Mechanical Heart Valve. Clin Med Insights Cardiol. 2016;10:11-7.
12. Bartczak-Rutkowska A, Trojnarska O, Cieplucha A, Markwitz W, Lesiak M. Maternal outcomes of pregnancy in women with mechanical heart valves prostheses - a single-center experience. Kardiol Pol. 2021;79(11):1262-4.
13. De Santo LS, Romano G, Della Corte A, D'Oria V, Nappi G, Giordano S, et al. Mechanical aortic valve replacement in young women planning on pregnancy: maternal and fetal outcomes under low oral anticoagulation, a pilot observational study on a comprehensive pre-operative counseling protocol. J Am Coll Cardiol. 2012;59(12):1110-5.

## Table S1. Data of interest for extraction

### Patient characteristics

Table S1. Overview of variables that were extracted from included studies

| **Variable** | **Data type** |
| --- | --- |
| Mean age at pregnancy | Numeric, continuous |
| Standard deviation of age at pregnancy | Numeric, continuous |
| Parity before surgery: nulliparous | Numeric, discrete |
| Parity before surgery: uniparous | Numeric, discrete |
| Parity before surgery: multiparous | Numeric, discrete |
| Parity before surgery: unknown | Numeric, discrete |
| Parity after surgery: primipara | Numeric, discrete |
| Parity after surgery: multipara | Numeric, discrete |
| Parity after surgery: unknown | Numeric, discrete |
| Number of patients on AC therapy | Numeric, discrete |
| Atrial fibrillation | Numeric, discrete |
| Congenital etiology | Numeric, discrete |
| Degenerative/ Calcific etiology | Numeric, discrete |
| Rheumatic etiology | Numeric, discrete |
| Endocarditis etiology | Numeric, discrete |
| Unknown etiology | Numeric, discrete |

### Procedural characteristics

| **Variable** | **Data** **type** |
| --- | --- |
| Prosthesis position: aortic | Numeric, discrete |
| Prosthesis position: mitral | Numeric, discrete |
| Prosthesis position: tricuspid | Numeric, discrete |
| Prosthesis position: pulmonary | Numeric, discrete |
| Prosthesis position: mitral + aortic | Numeric, discrete |
| Prosthesis position: pulmonary + aortic (ross) | Numeric, discrete |
| Prosthesis position: mitral + tricuspid | Numeric, discrete |
| Prosthesis position: unknown | Numeric, discrete |
| Prosthesis type: mechanical | Numeric, discrete |
| Prosthesis type: non-bi-leaflet mechanical valve | Numeric, discrete |
| Prosthesis type: bioprosthetic | Numeric, discrete |
| Prosthesis type: homograft | Numeric, discrete |
| Prosthesis type: autograft | Numeric, discrete |
| Prosthesis type: unknown | Numeric, discrete |
| Total number of valve prostheses implanted | Numeric, discrete |

### Pregnancy and maternal outcome

| **Variable** | **Data** **type** |
| --- | --- |
| Overall maternal mortality | Numeric, discrete |
| Cardiac maternal mortality | Numeric, discrete |
| Valve-related maternal mortality | Numeric, discrete |
| Maternal sudden unexplained death | Numeric, discrete |
| Pregnancy loss | Numeric, discrete |
| Stillbirth | Numeric, discrete |
| Miscarriage | Numeric, discrete |
| Termination: medical indication | Numeric, discrete |
| Termination: personal reason | Numeric, discrete |
| Fetal death due to preterm maternal death | Numeric, discrete |
| Valve reintervention | Numeric, discrete |
| Structural valve deterioration (SVD) | Numeric, discrete |
| Death due to SVD | Numeric, discrete |
| Reintervention due to SVD | Numeric, discrete |
| Non-structural valve deterioration (NSVD) | Numeric, discrete |
| Death due to NSVD | Numeric, discrete |
| Reintervention due to NSVD | Numeric, discrete |
| Endocarditis | Numeric, discrete |
| Death due to endocarditis | Numeric, discrete |
| Reintervention due to endocarditis | Numeric, discrete |
| Thromboembolic event | Numeric, discrete |
| Death due to thromboembolic event | Numeric, discrete |
| Valve thrombosis | Numeric, discrete |
| Death due to valve thrombosis | Numeric, discrete |
| Bleeding | Numeric, discrete |
| Death due to bleeding | Numeric, discrete |
| Mortality due to endocarditis | Numeric, discrete |
| Thromboembolism | Numeric, discrete |
| Valve thrombosis | Numeric, discrete |
| Mortality due to valve thrombosis | Numeric, discrete |
| Bleeding | Numeric, discrete |
| Obstetric bleeding | Numeric, discrete |
| Mortality due to bleeding | Numeric, discrete |
| Cerebrovascular accident (CVA) | Numeric, discrete |
| Ischemic CVA | Numeric, discrete |
| Hemorrhagic CVA | Numeric, discrete |
| Transient ischemic attack (TIA) | Numeric, discrete |
| Ischemic TIA | Numeric, discrete |
| Hemorrhagic TIA | Numeric, discrete |
| Maternal arrhythmia | Numeric, discrete |
| Pre-eclampsia | Numeric, discrete |
| Pregnancy hypertension | Numeric, discrete |
| Heart failure | Numeric, discrete |

### Study characteristic

| **Variable** | **Data** **type** |
| --- | --- |
| First author | Text |
| Year of publication | Text |
| Study design | Text |
| Number of patients | Numeric, discrete |
| Inclusion period start | Numeric, discrete |
| Inclusion period end | Numeric, discrete |
| Eligibility for subgroup: AC regimen A | Binary |
| Eligibility for subgroup: AC regimen B | Binary |
| Eligibility for subgroup: Mechanical prosthesis | Binary |
| Eligibility for subgroup: Biological prothesis | Binary |
| Antithrombotic regimen | Text |
| Antithrombotic drug agent | Text |
| INR range min | Numeric, continuous |
| INR range max | Numeric, continuous |

### Fetal and delivery outcomes

| **Variable** | **Data** **type** |
| --- | --- |
| Vaginal birth | Numeric, discrete |
| Postpartum bleeding | Numeric, discrete |
| Caesarean section | Numeric, discrete |
| Postoperative bleeding | Numeric, discrete |
| Delivered alive | Numeric, discrete |
| Perinatal mortality | Numeric, discrete |
| Neonatal mortality | Numeric, discrete |
| Small for gestational age | Numeric, discrete |
| Low birthweight | Numeric, discrete |
| Preterm birth | Numeric, discrete |
| Congenital malformation | Numeric, discrete |
| Hydrocephalus | Numeric, discrete |
| Respiratory distress syndrome | Numeric, discrete |
| Admission NICU | Numeric, discrete |
| Anticoagulant embryopathy | Numeric, discrete |

## Table S2

| **Study** | **Year of publication** | **Country** | **Income country?** | **Study type** | **Mean year of inclusion** | **Mean age at pregnancy** | **Number of patients** | **Number of pregnancies** |
| --- | --- | --- | --- | --- | --- | --- | --- | --- |
| Sadler et al. | 2000 | New Zeeland | High | Retrospective | 1982 | 26.1 | 21 | 34 |
| Yap et al. | 2007 | Netherlands & Belgium | High | Retrospective | 2005 | 29.6 | 5 | 12 |
| De Santo et al. | 2012 | Italy | High | Prospective | 2005 | 26.9 | 17 | 17 |
| Heuvelman et al. | 2013 | Netherlands | High | Retrospective | 1999 | 29.3 | 40 | 67 |
| Basude et al. | 2014 | United Kingdom (Bristol) | High | Retrospective | 2005 | 29.4 | 16 | 32 |
| Bouhout et al. | 2014 | Canada (Montreal) | High | Prospective | 1995 | - | 14 | 27 |
| Morimoto et al. | 2015 | Japan | High | Retrospective | 1999 | - | 8 | 11 |
| Ayad et al. | 2016 | Egypt | Low-middle | Prospective | - | - | 22 | 22 |
| Carvajal et al. | 2018 | United States | High | Retrospective | 2003 | 25.5 | 11 | 23 |
| Bartczak et al. | 2021 | Poland | High | Retrospective | 2010 | 28.7 | 10 | 13 |
| Wichert et al. | 2022 | Canada (CARPREG) | High | Retrospective | 2006 | 31 | 38 | 42 |
| Thompson et al. | 2023 | United Kingdom (Birmingham) | High | Retrospective | 2009 | 23.9 | 12 | 19 |
| Nappi et al | 2023 | France | High | Retrospective | 2001 | - | 37 | 37 |
| **Total** |  |  |  |  |  |  | **251** | **356** |

## Table S3 PRISMA Guidelines Checklist

### Abstract Prisma 2020 Checklist

| **Section and Topic** | **Item #** | **Checklist item** | **Reported (Yes/No)** |
| --- | --- | --- | --- |
| **TITLE** | | |  |
| Title | 1 | Identify the report as a systematic review. | ✅ |
| **BACKGROUND** | | |  |
| Objectives | 2 | Provide an explicit statement of the main objective(s) or question(s) the review addresses. | ✅ |
| **METHODS** | | |  |
| Eligibility criteria | 3 | Specify the inclusion and exclusion criteria for the review. | ✅ |
| Information sources | 4 | Specify the information sources (e.g. databases, registers) used to identify studies and the date when each was last searched. | ✅ |
| Risk of bias | 5 | Specify the methods used to assess risk of bias in the included studies. | ✅ |
| Synthesis of results | 6 | Specify the methods used to present and synthesise results. | ✅ |
| **RESULTS** | | |  |
| Included studies | 7 | Give the total number of included studies and participants and summarise relevant characteristics of studies. | ✅ |
| Synthesis of results | 8 | Present results for main outcomes, preferably indicating the number of included studies and participants for each. If meta-analysis was done, report the summary estimate and confidence/credible interval. If comparing groups, indicate the direction of the effect (i.e. which group is favoured). | ✅ |
| **DISCUSSION** | | |  |
| Limitations of evidence | 9 | Provide a brief summary of the limitations of the evidence included in the review (e.g. study risk of bias, inconsistency and imprecision). | ✅ |
| Interpretation | 10 | Provide a general interpretation of the results and important implications. | ✅ |
| **OTHER** | | |  |
| Funding | 11 | Specify the primary source of funding for the review. | ✅ |
| Registration | 12 | Provide the register name and registration number. | ✅ |

### Full text Prisma 2020 Checklist

| **Section and Topic** | **Item #** | **Checklist item** | **Location where item is reported** |
| --- | --- | --- | --- |
| **TITLE** | | |  |
| Title | 1 | Identify the report as a systematic review. | ✅ |
| **ABSTRACT** | | |  |
| Abstract | 2 | See the PRISMA 2020 for Abstracts checklist. | ✅ |
| **INTRODUCTION** | | |  |
| Rationale | 3 | Describe the rationale for the review in the context of existing knowledge. | Line 87-93 |
| Objectives | 4 | Provide an explicit statement of the objective(s) or question(s) the review addresses. | Line 94-999 |
| **METHODS** | | |  |
| Eligibility criteria | 5 | Specify the inclusion and exclusion criteria for the review and how studies were grouped for the syntheses. | Protocol and inclusion criteria |
| Information sources | 6 | Specify all databases, registers, websites, organisations, reference lists and other sources searched or consulted to identify studies. Specify the date when each source was last searched or consulted. | Line 117/118 |
| Search strategy | 7 | Present the full search strategies for all databases, registers and websites, including any filters and limits used. | Supplementary Text S1 |
| Selection process | 8 | Specify the methods used to decide whether a study met the inclusion criteria of the review, including how many reviewers screened each record and each report retrieved, whether they worked independently, and if applicable, details of automation tools used in the process. | Search and study selection |
| Data collection process | 9 | Specify the methods used to collect data from reports, including how many reviewers collected data from each report, whether they worked independently, any processes for obtaining or confirming data from study investigators, and if applicable, details of automation tools used in the process. | Data extraction |
| Data items | 10a | List and define all outcomes for which data were sought. Specify whether all results that were compatible with each outcome domain in each study were sought (e.g. for all measures, time points, analyses), and if not, the methods used to decide which results to collect. | Line 128-133, Supplementary Table 1 |
|  | 10b | List and define all other variables for which data were sought (e.g. participant and intervention characteristics, funding sources). Describe any assumptions made about any missing or unclear information. | Line 134-143 |
| Study risk of bias assessment | 11 | Specify the methods used to assess risk of bias in the included studies, including details of the tool(s) used, how many reviewers assessed each study and whether they worked independently, and if applicable, details of automation tools used in the process. | Line 126, 161-163 Supplementary table S7 |
| Effect measures | 12 | Specify for each outcome the effect measure(s) (e.g. risk ratio, mean difference) used in the synthesis or presentation of results. | Table 2, Table 3 |
| Synthesis methods | 13a | Describe the processes used to decide which studies were eligible for each synthesis (e.g. tabulating the study intervention characteristics and comparing against the planned groups for each synthesis (item #5)). | Line 106-115, Figure 1 |
|  | 13b | Describe any methods required to prepare the data for presentation or synthesis, such as handling of missing summary statistics, or data conversions. | Line 127, 145-159 |
|  | 13c | Describe any methods used to tabulate or visually display results of individual studies and syntheses. | Line 153-154 |
|  | 13d | Describe any methods used to synthesize results and provide a rationale for the choice(s). If meta-analysis was performed, describe the model(s), method(s) to identify the presence and extent of statistical heterogeneity, and software package(s) used. | Line 150-159 |
|  | 13e | Describe any methods used to explore possible causes of heterogeneity among study results (e.g. subgroup analysis, meta-regression). | Line 150-159, Table 2, Table 3 |
|  | 13f | Describe any sensitivity analyses conducted to assess robustness of the synthesized results. | Line 161-163 |
| Reporting bias assessment | 14 | Describe any methods used to assess risk of bias due to missing results in a synthesis (arising from reporting biases). | Line 153-154, 162-163 |
| Certainty assessment | 15 | Describe any methods used to assess certainty (or confidence) in the body of evidence for an outcome. | Line 150-159, Table 3 |
| **RESULTS** | | |  |
| Study selection | 16a | Describe the results of the search and selection process, from the number of records identified in the search to the number of studies included in the review, ideally using a flow diagram. | Figure 1 |
|  | 16b | Cite studies that might appear to meet the inclusion criteria, but which were excluded, and explain why they were excluded. | Line 110-113 |
| Study characteristics | 17 | Cite each included study and present its characteristics. | Table 1, Supplementary Text S2 and Table S2 |
| Risk of bias in studies | 18 | Present assessments of risk of bias for each included study. | Line 259,  Table S7 |
| Results of individual studies | 19 | For all outcomes, present, for each study: (a) summary statistics for each group (where appropriate) and (b) an effect estimate and its precision (e.g. confidence/credible interval), ideally using structured tables or plots. | Table 2, Table 3, Figure 2a-d, Figure 3a-d |
| Results of syntheses | 20a | For each synthesis, briefly summarise the characteristics and risk of bias among contributing studies. | Supplementary table S7 |
|  | 20b | Present results of all statistical syntheses conducted. If meta-analysis was done, present for each the summary estimate and its precision (e.g. confidence/credible interval) and measures of statistical heterogeneity. If comparing groups, describe the direction of the effect. | Table 2, Table 3 |
|  | 20c | Present results of all investigations of possible causes of heterogeneity among study results. | Table 2, Table 3 |
|  | 20d | Present results of all sensitivity analyses conducted to assess the robustness of the synthesized results. | Line 257-266  Supplementary Materials  Tables S4, S5, S6 and Figures S1a-1d |
| Reporting biases | 21 | Present assessments of risk of bias due to missing results (arising from reporting biases) for each synthesis assessed. | Strengths and Limitations. Supplementary Table S4 & Table S5 |
| Certainty of evidence | 22 | Present assessments of certainty (or confidence) in the body of evidence for each outcome assessed. | Table 2, Table 3 |
| **DISCUSSION** | | |  |
| Discussion | 23a | Provide a general interpretation of the results in the context of other evidence. | Discussion, Strengths & Limitations |
|  | 23b | Discuss any limitations of the evidence included in the review. | Strengths and Limitations,  Line 382-412 |
|  | 23c | Discuss any limitations of the review processes used. | Strengths and Limitations |
|  | 23d | Discuss implications of the results for practice, policy, and future research. | Lines 281-284, 305-310, 372-381 |
| **OTHER INFORMATION** | | |  |
| Registration and protocol | 24a | Provide registration information for the review, including register name and registration number, or state that the review was not registered. | Cover page, methods.  Sub-study of PROSPERO CRD-42015017041 |
|  | 24b | Indicate where the review protocol can be accessed, or state that a protocol was not prepared. | Cover page, line 105 |
|  | 24c | Describe and explain any amendments to information provided at registration or in the protocol. | Methods (line 104) |
| Support | 25 | Describe sources of financial or non-financial support for the review, and the role of the funders or sponsors in the review. | Disclosures |
| Competing interests | 26 | Declare any competing interests of review authors. | Disclosures |
| Availability of data, code and other materials | 27 | Report which of the following are publicly available and where they can be found: template data collection forms; data extracted from included studies; data used for all analyses; analytic code; any other materials used in the review. | Upon request to corresponding author |

## Table S4 – Sensitivity Analysis Baseline Characteristics – exclusion outliers for time-to-pregnancy interval

| Sensitivity analysis | Autograft | Homograft | Bioprosthesis | Mechanical |
| --- | --- | --- | --- | --- |
| Pooled mean time from valve replacement to pregnancy, y±SD | 8.6 ± 5.2 | 4.12 ± 3.3 | 5.9 ± 2.9 | 10.3 ± 5.8 |

Table S5 – Pooled reintervention rates during pregnancy with the limited data available

|  | **Autograft** | **Homograft** | **Bioprosthesis** | **Mechanical** |
| --- | --- | --- | --- | --- |
| **N studies reporting the outcome** | 4 | 1 | 4 | 1 |
| **N patient-years** | 298 | 469 | 38,6 | 26.5 |
| **N Events** | 0 | 0 | 1 | 4 |
| **Pooled incidence rate** | N/A* | N/A* | 7.1%/py (95%CI: 2.04-24.4) | 2.08%/py (95%CI: 0.13-33.3) |

*****for these variables, RStudio uses an automatic continuity correction as all studies have 0-events, resulting in highly unreliable estimations.

95% CI = 95% Confidence interval

## Table S6 Results Sensitivity Analysis / Exclusion Lower-Middle income country

|  | **Total population** | **I^2^** | **n** |  | **Mechanical** | **I^2^** | **n** |
| --- | --- | --- | --- | --- | --- | --- | --- |
| Maternal outcomes |  |  |  |  |  |  |  |
| Maternal death | 0.23 (0.0 -50.3) | 0 | 11 |  | 1.5 (0.2-10.0) | 0 | 5 |
| Valve related reintervention (excl reintervention for VT) | 1.1 (0.3-4.3) | 0 | 10 |  | 0 | 0 | 5 |
| Valve thrombosis (VT) | 1.5 (0.3-8.9) | 0 | 10 |  | 6.1 (1.9-45.5) | 0 | 5 |
| Reintervention for VT | - | - | 0 |  | 6.8 (2.6-16.7) | 0 | 4 |
| Other thrombo-embolic event | 1.7 (0.5-5.1) | 0 | 10 |  | 4.6 (1.5-13.7) | 0 | 5 |
| Permanent decrease AI | 1.3 (0.02-45.1) | 0 | 6 |  | 0 | 0 | 2 |
| Pregnancy outcomes |  |  |  |  |  |  |  |
| Delivered alive* | 90.8 (77.4-96.6) | 63% (p = 0.00) | 13 |  | 74.5 (44.2-91.5) | 21.8 | 5 |
| Miscarriage* | 4.1 (1.0-15.1) | 51% (p = 0.02) | 13 |  | 16.6 (3.6 -51.5) | 22.1 | 4 |
| Stillbirth* | 1.3 (0.5-3.4) | 0% (p = 1.00) | 11 |  | 1.9 (0.2-12.2) | 0 | 4 |
| Abortion (maternal indication) * | 1.6 (0.6-4.2) | 0% (p = 1.00) | 9 |  | 7.8 (0.8-22.4) | 0 | 4 |
| Bleeding * | 4.3 (1.7 - 10.7) | 40% (p= 0.09) | 10 |  | 5.64 (1.55-18.51) | 0 | 5 |
| Heart failure during pregnancy* | 2.6 (0.9-7.6) | 0% (p = 0.99) | 10 |  | 1.5 (0.02-53.8) | 0 | 4 |
| Pregnancy hypertension* | 4.4 (2.1-8.9) | 0% (p = 0.95) | 5 |  | 0 | 0 | 3 |
| Pre-eclampsia* | 1.3 (0.3-4.9) | 0% (p = 1.00) | 5 |  | - | - | 0 |
| Pre-term birth* | 8.3 (4.2-15.7) | 0% (p = 0.80) | 10 |  | 10.5 (4.8-21.5) | 0 | 5 |
| Low birth weight* | 6.2 (2.6-14.0) | 20% (p = 0.29) | 3 |  | 0 | 0 | 2 |
| Small for gestational age* | 5.3 (1.5 - 16.7) | 0% (p = 0.58) | 6 |  | 0 | 0 | 3 |
| Neonatal death* | 0.8 (0.1-6.5) | 0% (p = 0.92) | 10 |  | 0 | 0 | 4 |

## Table S7 Quality assessment Newcastle-Ottawa Scale Cohort Studies

| Study, (year) | Selection | Comparability | Outcome |
| --- | --- | --- | --- |
| Sadler et al. (2000) | 3 | 0 | 2 |
| Yap et al. (2007) | 3 | 0 | 3 |
| De Santo et al.(2012) | 3 | 0 | 2 |
| Heuvelman et al. (2013) | 3 | 0 | 3 |
| Basude et al. (2014) | 3 | 0 | 3 |
| Bouhout et al.(2014) | 3 | 0 | 3 |
| Morimoto et al. (2015) | 3 | 0 | 3 |
| Ayad et al. (2016) | 2 | 0 | 1 |
| Carvajal et al.(2018) | 3 | 0 | 3 |
| Bartczak et al. (2021) | 2 | 0 | 3 |
| Wichert et al. (2022) | 3 | 0 | 3 |
| Thompson et al. (2023) | 3 | 0 | 3 |
| Nappi et al. (2023) | 2 | 0 | 1 |

## Supplementary Figure S1 - Forest plots low-middle income country excluded

A: Valve thrombosis


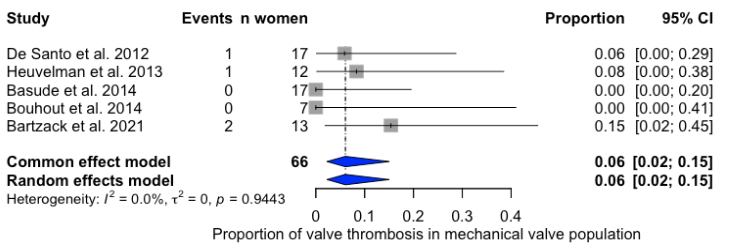


B: Other ischemic events

##
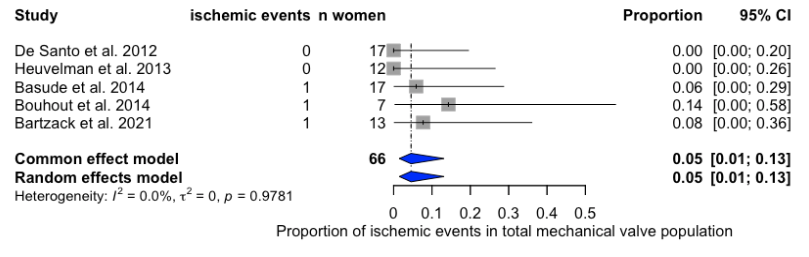


C: Obstetric hemorrhage
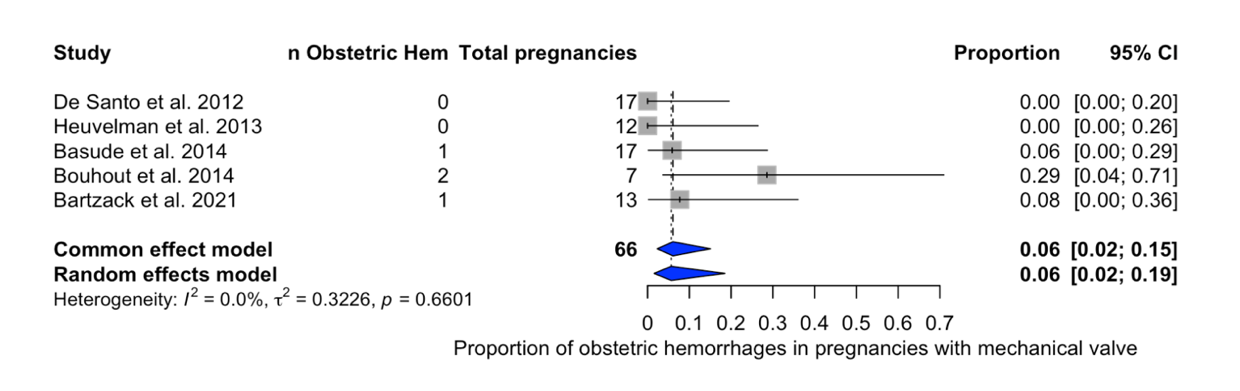


D: Miscarriage


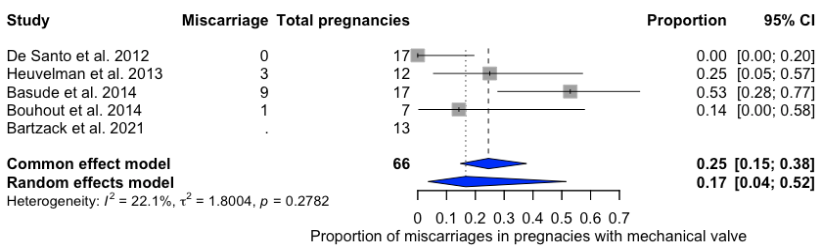

Supplement: ezag137_Supplementary_Data [file ezag137_supplementary_data.docx]
